# Tactile information from the vibrissal system modulates hippocampal functioning

**DOI:** 10.1016/j.crneur.2022.100034

**Published:** 2022-03-11

**Authors:** Nereida Ibarra-Castaneda, Norma A. Moy-Lopez, Oscar Gonzalez-Perez

**Affiliations:** a Laboratory of Neuroscience, School of Psychology, University of Colima, Colima, Mexico; b Medical Science PhD Program, School of Medicine, University of Colima, Colima, Mexico

**Keywords:** Vibrissae, Hippocampus, Barrel cortex, Adult neurogenesis, Subgranular zone

## Abstract

Most mammals have sensory tactile hairs, also known as whiskers or vibrissae. Traditionally, whiskers are associated with diverse survival skills, including tactile discrimination, distance assessment, food acquisition, gap crossing, and social interaction. Vibrissae functions are processed in the somatosensorial cortex, commonly referred to as the barrel cortex. Hence, most of the whisker-related research has been focused on this cortical region. However, increasing evidence indicates that the vibrissal system modulates several aspects of hippocampal physiology. This graphical review aims to summarize cumulative evidence indicating that whiskers regulate the neural function and cellularity in several hippocampal subfields. Interestingly, lack of whiskers notably affects neuronal firing in CA1 and CA3 hippocampal subfields, alters spatial mapping, impairs navigational skills, modifies cytoarchitecture, and reduces the adult neurogenesis in the dentate gyrus. This evidence extends our understanding of how whiskers are related to hippocampal function and offers insights to explore novel associations between whisker functions and neural plasticity in the hippocampus.

## Highlights

• Whiskers provide tactile information to the somatosensory cortex and hippocampus.
• Lack of whiskers reduces neuronal firing in CA1 and CA3 hippocampal subfields.
• Whisker activity modulates spatial mapping, navigational skills, and c-Fos expression.
• Whisker deprivation reduces the adult neurogenesis in the dentate gyrus.

## Graphical abstract

**Figure:**
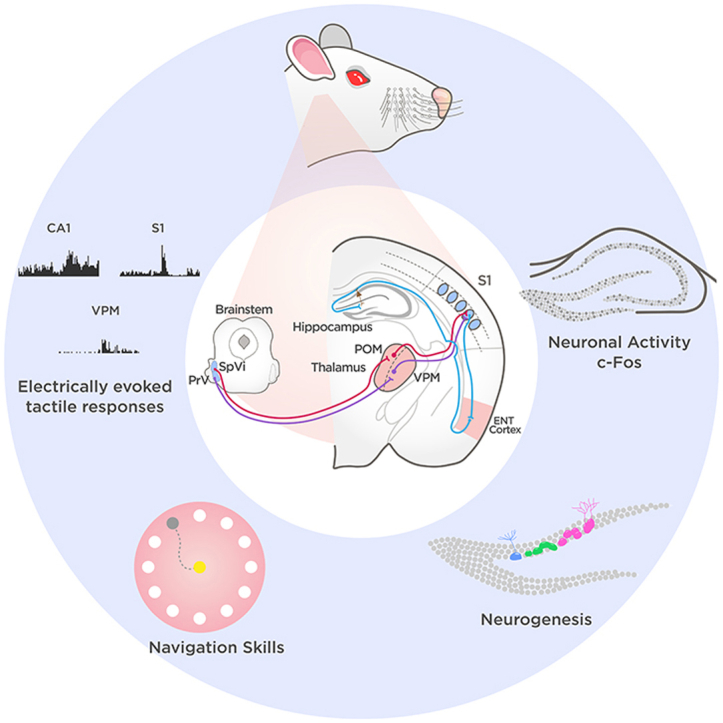

## 1 Introduction

**Fig. 1:**
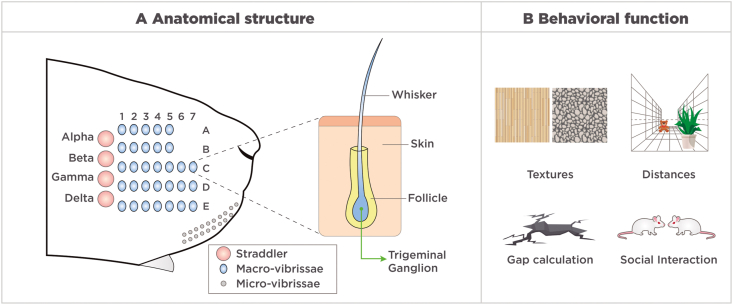
Representation of the vibrissae system and its function. **(A)** Anatomical structure of vibrissae. The structure that anchors each whisker to the skin is called follicle, which gives tactile sensitivity and movement to whiskers. Each follicle has mechanoreceptors and nerve terminals that respond specifically to whisker movement and the tactile information from each follicle is transported via the infraorbital nerve (ganglion trigeminal). Vibrissal system comprises micro-vibrissae (short and thin hairs located at the tip of the nose) and mystical macro-vibrissae (long and stiff hairs located on the vibrissae pad). Macro-vibrissae consist of four follicles arranged in rows A and B, 7 to 9 follicles in rows C, D and E, ranging from 15 to 50 mm. Straddles are the longest macro-vibrissae located in the most caudal part of the vibrissae pad, which are designated as alpha, beta, gamma and delta (Adibi, 2019; Brecht et al., 1997; Voges et al., 2012) (**B)** Behavioral functions of vibrissal system. Rodents use whiskers to obtain information about the texture and distance of objects, calculate gap widths, and develop social interaction. Animals collect the information through an active process called "whisker beating" (a back-and-forth movement of whiskers) to identify the characteristics of an object (Deschênes et al., 2005; Diamond et al., 2008).

Rodents require exquisite navigational skills for environment exploration and, many of these abilities rely on their vibrissal system, also referred to as whiskers or facial hairs (Fig. 1A). Traditionally, whiskers are associated with tactile discrimination, gap crossing, and social behavior (Fig. 1B) (Adibi, 2019; Ahl, 1986; Brecht et al., 1997). However, increasing evidence indicates that the vibrissal system regulates hippocampal function, cytoarchitecture, and neurogenesis (Gonzalez-Perez et al., 2018; Milshtein-Parush et al., 2017; Pereira et al., 2007).

Whiskers are arranged in a five-row pad on each side of rodents’ snout and each row pad contains between five and nine whiskers 15–50 mm long (Fig. 1A). Whiskers are classified in *macro-vibrissae*, long hairs responsible for general object scanning and spatial navigation, and *micro-vibrissae,* short hairs responsible for exploring the specific properties of objects and texture recognition. Both types of whiskers work together to help animals with tactile discrimination, distance assessment, food acquisition, gap crossing, and social behavior (Fig. 1B) (Deschênes et al., 2005; Diamond et al., 2008). All whiskers are connected to structures, named follicles, innervated by the infraorbital nerve (**ION**) and, each follicle has mechanoreceptors that respond to the movement of whiskers to provide tactile sensitivity (Fig. 1A) (Diamond et al., 2008).

**Fig. 2:**
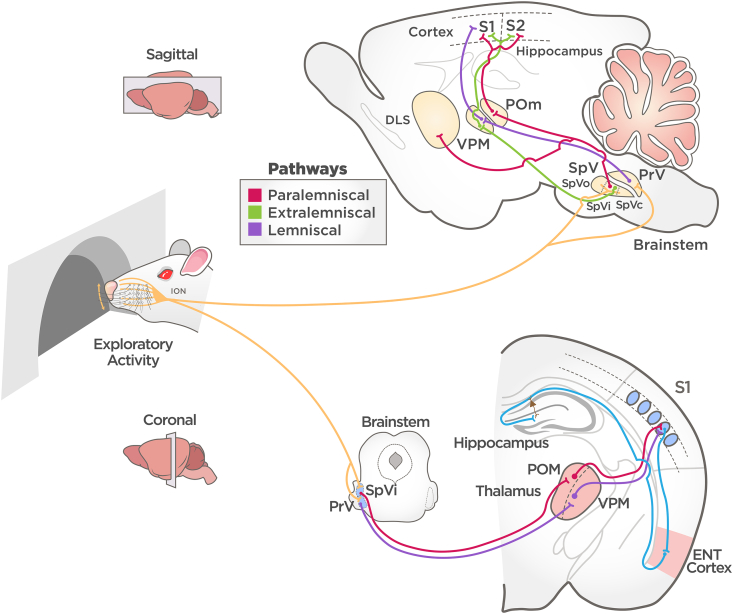
Sagittal and coronal views of the trigeminal-thalamocortical vibrissae pathways. A) Sagittal view: Vibrissae flexion evokes action potentials in trigeminal ganglion neurons. In this region, the tactile information sensed by whiskers is processed in two sensory nuclei: the principal sensory nucleus (**PrV**) and the spinal nucleus (**SpV**) that, in turn, is subdivided into 3 sub-nuclei: oralis (**SpVo**), interpolaris (**SpVi**), and caudalis (**SpVc**). Following, tactile information is conducted to the cortex through three pathways: lemniscal, paralemniscal, and extralemniscal. The lemniscal pathway derives from the PrV and is the main pathway that carries tactile signals to the cortex. In the PrV, neuronal groups, called barrelettes, preserve the somatotopic organization of vibrissae in the brainstem. Most of the barrelette neurons project to the ventral posteromedial nucleus **(VPM)** in the contralateral thalamus that, in turn, project to the layer IV of the somatosensory cortex S1 in cortical barrel columns. The paralemniscal pathway carries sensory information from the SpVi to the posterior medial nucleus of the thalamus **(POM)** that, in turn, relays to the S1 and S2 cortical regions and the dorsolateral striatum (**DLS**). This pathway transmits positional reference and sensorimotor coordination signals during vibrissae flexion (Ahissar et al., 2000). The extralemniscal pathway transmits tactile information from the caudal division of SpVi and SpVo, and then projects to the VPM, and then to S2 and septal regions of S1 cortex. Coronal view: Tactile information processed in the somatosensory cortex projects to the lateral entorhinal cortex (**ENT**) via indirect projections through the perirhinal cortex. This potentially forms an additional pathway of vibrissal information to target the hippocampus through the lemniscal pathway (Adibi, 2019; Aronoff et al., 2010; Le Merre et al., 2018).

The tactile information obtained from vibrissal follicles projects via the ION to the principal sensory nucleus (**Prv**) and the spinal nucleus (**SpV**) (Fig. 2). After synapsing in the brainstem, axons from second-order neurons cross the midline and reach the contralateral ventral posteromedial nucleus (**VPM**) of the thalamus through the lemniscal pathway (Bisler et al., 2002; Diamond et al., 2008; Frangeul et al., 2014). Then, thalamic neurons project to layer IV of the somatosensory cortex, commonly known as the barrel cortex (**BC**) (Bisler et al., 2002). Subsequently, some of this tactile information processed in the somatosensory cortex projects to the entorhinal cortex **(ENT)** to finally reach the hippocampus (Fig. 2). Thus, the active use of whiskers elicits neuronal activity that promotes neuronal plasticity in the BC and helps create spatial maps in the adult hippocampus.

**Fig. 3:**
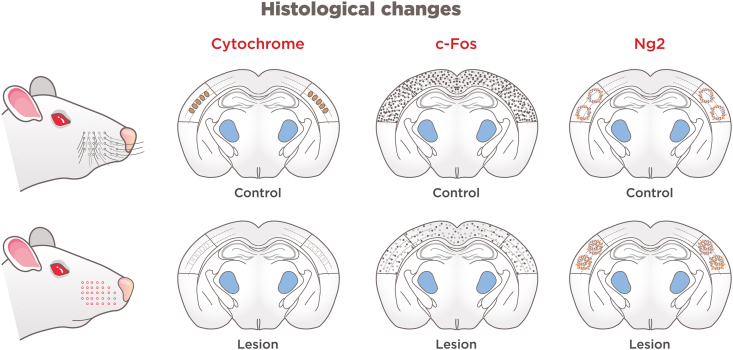
Histological changes in sensory deprivation. During vibrissae removal, changes in different neuronal markers can occur. The enzymatic activity (cytochrome oxidase) decreases drastically, and the typical circular shape of the barrels disappears in the whisker-deprived group when compared to controls. The proto-oncogene c-Fos, an immediate early gene and marker of neuronal activity, is expressed in response to diverse stimuli. The expression of c-Fos decreases markedly in whisker-deprived animals, whereas the expression of c-Fos increases after whisker stimulation. Electrocauterization of vibrissae produces high proliferation rates of NG2 glial cells in the core of barrels (Dimou and Götz, 2012; Filipkowski et al., 2001; Killackey et al., 1994).

In the BC, each vibrissal follicle has a precise neuronal representation (Ahl, 1986; Grion et al., 2016). Each barrel processes information from a single whisker and, all of them, integrate a cortical map that replicates the vibrissal pad (Dimou and Götz, 2012). During vibrissae stimulation, every barrel responds rapidly and modulates its neural activity depending on the nature of the stimulus (Woolsey and Van der Loos, 1970). Thus, vibrissae elimination produces a significant decrease in the activity of cytochrome oxidase **(COX)** enzyme in the BC (Fig. 3) (Killackey et al., 1994; Wong-Riley and Welt, 1980). Whisker deprivation also produces a dramatic decrease in the expression of a calcium-dependent marker for neuronal activity (c-Fos protein) (Perrin-Terrin et al., 2016) in the BC (Filipkowski et al., 2001; Gonzalez-Perez et al., 2018). In contrast, tactile experience or stimulation enhances the c-Fos expression (Fig. 3) (Bisler et al., 2002; Filipkowski et al., 2000; Lecrux et al., 2017). Remarkably, some glial cells in the BC are also modulated by tactile stimuli. Synaptic inputs from thalamocortical fibers derived from the vibrissal system regulate the number and proliferation of NG2 glia in the BC, which confirms that tactile inputs strongly modulate the neural activity of this region (Dimou and Götz, 2012). Thus, research on sensorial information in the BC is extensive, but emerging evidence indicates that sensorial experience can modulate hippocampal function.

**Fig. 4:**
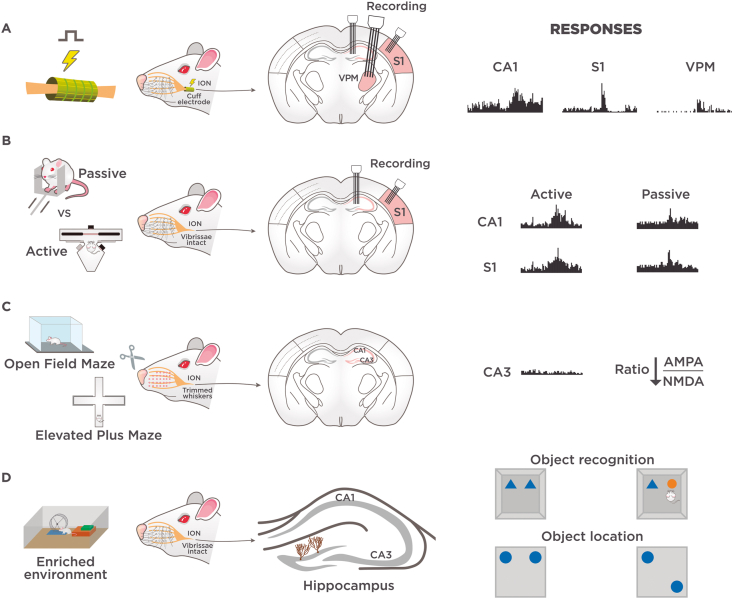
Electrically evoked tactile responses in the hippocampus (CA1 region), primary somatosensory cortex (**S1**), and ventral posteromedial nucleus (**VPM**). **(A)** Cuff electrodes (green cylinders) were placed on the infraorbital nerve (ION) to produce electrical stimulation; simultaneously, multielectrode arrays of micro-wires (black figures) were implanted in the CA1, VPM, and S1 to record the responses elicited from cuff-electrode stimulation. Thus, 52% CA1 cells, 49% S1 cells, and 32% VPM cells responded to this electrical stimulation. This evidence indicated that tactile inputs reached the CA1 hippocampal region through the VPM and S1. (Pereira et al., 2007). **(B)** Rodents were exposed to two independent stimulation protocols. In the first protocol, the animal's head is fixed while whiskers are stimulated by passing the nose through a sliding aperture (passive stimulation). In the second protocol, the animal was allowed to move freely (active whisker stimulation) during a multielectrode recording in the CA1 and S1 regions. Robust neuronal responses were observed in the CA1 hippocampal region, predominantly during active stimulation (Pereira et al., 2007). (C) Conversely, repetitive shear removal of vibrissae during the early stages of brain development suppresses the neuronal activity of CA3 pyramidal neurons and decreases the probability of vesicle releasing, an event that promotes CA1-CA3 synaptic facilitation. This type of whisker deprivation affects the probability of glutamate releasing and reduces the AMPA/NMDA ratio, suggesting that changes in AMPA-mediated synaptic transmission are linked to an increase in the expression of NMDA receptors. Hence, sensory deprivation interferes with the maturation of AMPA/NMDA synaptic receptors in the hippocampus (Milshtein-Parush et al., 2017). These synaptic changes in the dorsal hippocampus seem not to be related to deprivation-induced stress as observed by the animal performance in the elevated cross-field and open-field mazes (Milshtein-Parush et al., 2017). **(D)** Tactile enrichment increases the dendritic length and density of spines in DG neurons. Interestingly, the activation of dorsal and ventral DG neurons by chemogenetic manipulation significantly improves object recognition and object location tasks, respectively (Wang et al., 2020). Altogether, this evidence indicates that facial vibrissae are sensory organs used as high-resolution tactile discriminators for the dynamic updating of spatial maps in the adult hippocampus.

**Fig. 5:**
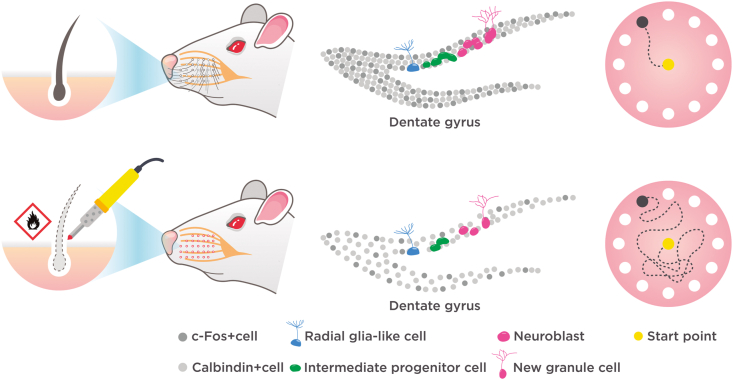
Tactile information from whiskers regulates hippocampal neurogenesis and cellular homeostasis in the dentate gyrus (**DG**). Whisker removal by electrocauterization produces a depletion of calbindin and c-Fos protein in the DG, which are accompanied by a significant reduction in the hippocampal neurogenesis by reducing the total number of intermediate progenitor cells (Sox2-expressing cells), neuroblast (doublecortin-expressing cells), and new granulate neurons (NeuN+ cells) in the subgranular zone of the DG. These events seem to impair navigational skills to solve a spatial memory task in the Barnes' maze (Gonzalez-Perez et al., 2018).

The hippocampus is an essential region for contextual learning, memory consolidation, cognition, and novelty detection, which requires the integration of sensorial systems (visual, olfactory, and tactile) (Fig. 4 A – C) (Milshtein-Parush et al., 2017; Pereira et al., 2007; Zeidman and Maguire, 2016). Electrically evoked tactile responses recorded in the CA1 hippocampus indicate that tactile inputs reach this region via the somatosensory cortex and contribute to acquire experience-dependent learning (Le Merre et al., 2018). Interestingly, trigeminal inputs from the vibrissae system send projections to CA1 and CA3 hippocampal regions and dentate gyrus **(DG)** to process this information and modulate neuronal spiking activity during discrimination tasks (Lee et al., 2004). A recent report indicates that tactile experience enrichment improves memory and alleviates anxiety by inducing neuronal remodeling along the dorsoventral axis of the DG (Wang et al., 2020), which demonstrates that whisker activity strongly regulates the neuronal functioning in this neurogenic region (Fig. 4 D). In this regard, voluntary exercise, enriched environments, and cognitive processes can change hippocampal functioning in an activity-dependent manner that, in turn, can modify the adult neurogenesis (Kempermann et al., 1997; Ma et al., 2009; Pereira et al., 2007). Notably, whisker elimination reduces the number of c-Fos+ cells in the CA1, CA2, and CA3 hippocampal subfields and decreases calbindin expression in the DG. Intriguingly, these events are linked to a substantial reduction in the hippocampal neurogenesis, which affects the total number of new neurons produced in the adult subgranular zone **(SGZ)** and disrupts navigational skills that help solve a spatial memory task (Fig. 5) (Gonzalez-Perez et al., 2018). Altogether, these findings unveiled an important neurophysiological interaction among tactile information, hippocampal neurogenesis, and the creation of spatial maps in the postnatal brain. The biological meaning of this interaction is not clear, but recent evidence indicates that whisker-related sensory information is processed in the hippocampus to produce contextual, experience-dependent learning of rewarded sensory-motor associations (Le Merre et al., 2018). Thus, sensory responses elicited during contextual learning of goal-directed behaviors require the interaction among several cortical areas (prefrontal, entorhinal, S1, and S2 cortices) with the hippocampal circuitry (CA1 and CA3) and its neurogenic niche (Adibi, 2019; Aronoff et al., 2010; Cameron and Glover, 2015; Le Merre et al., 2018). Altogether, this evidence suggests that the tactile information processed in the hippocampus contributes to the learning of rewarded sensory-motor associations.

In summary, the tactile information processed through the vibrissal system is a crucial regulator of hippocampal functioning, neuronal remodeling, and neurogenesis, which unveils new associations between whisker activity and hippocampal homeostasis. Knowing the relationship between sensorial deprivation/stimulation and hippocampal plasticity may help understand processes of associative learning, cognitive decline, mental disorders, and neurodegeneration observed after long-lasting sensory deprivation.

## CRediT authorship contribution statement

**Nereida Ibarra-Castaneda:** First draft, Writing – original draft, Formal analysis, figure design. **Norma A. Moy-Lopez:** Formal analysis. **Oscar Gonzalez-Perez:** Conception of the work, figure design, Writing – review & editing, final approval of the work.

## Declaration of competing interest

The authors declare that they have no known competing financial interests or personal relationships that could have appeared to influence the work reported in this paper.
