# Supplementary material for: Tactile information from the vibrissal system modulates hippocampal functioning
Source: Curr Res Neurobiol. 2022 Mar 11;3:100034. doi: 10.1016/j.crneur.2022.100034 (PMC9140216; doi:10.1016/j.crneur.2022.100034)
Supplement: Multimedia component 1 [file mmc1.pdf]

Peer Review Overview

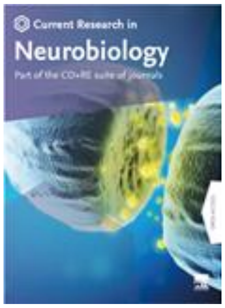

Manuscript Title: Tactile information from the vibrissal system modulates hippocampal functioning

Received	Oct 26,2021
1st Decision	Dec 06,2021
1st Revision Submitted	Jan 28,2022
Accepted	Feb 24,2022

1st Decision letter

Reference: CRNEUR-D-21-00062

Title: Tactile information from the vibrissal system modulates hippocampal functions

Journal: Current Research in Neurobiology

Dear Dr. Gonzalez-Perez,

Thank you for submitting your manuscript to Current Research in Neurobiology.

I have completed my evaluation of your manuscript. The reviewers recommend reconsideration of your manuscript following revision. I invite you to resubmit your manuscript after addressing the comments below. Please resubmit your revised manuscript by Jan 31, 2022.

When revising your manuscript, please consider all issues mentioned in the reviewers' comments carefully: please outline in a cover letter every change made in response to their comments and provide suitable rebuttals for any comments not addressed. Please note that your revised submission may need to be re-reviewed.

Current Research in Neurobiology values your contribution and I look forward to receiving your revised manuscript.

CRNEUR aims to be a unique, community-led journal, as highlighted in the Editorial Introduction. As part of this vision, we will be regularly seeking input from the scientific community. We encourage you and your co-authors to take the survey as part of the editorial.

Kind regards,

Abhishek Banerjee
Associate Editor
Current Research in Neurobiology

Comments from Editors and Reviewers:

Reviewer #1:

The manuscript provides a timely review of the processing of whisker-mediated sensory information in the hippocampus and modulation of hippocampal cytoarchitecture and neurogenesis through vibrissae-based sensory experience or deprivation. The figures are visually appealing, and the authors start by providing an overview of the well-described pathways that convey whisker information to sensory cortex and observed sensory-experience dependent changes in the sensory cortex. Then, they highlight recent findings regarding the changes in hippocampus function and structure after whisker-mediated sensory experiences, putting these findings in a more global context. The clear synthesis and summarized schematic representations of the results will be of great use for researchers whishing to learn more about this interesting body of work.
However, I believe the manuscript should be heavily improved. Most importantly, some statements are not supported by clear references and other references are not described correctly (see below for examples). For more detailed comments, please find below my two major and a few minor suggestions:

Major comment:
1. The authors claim in the text (e.g. page 3, paragraph 3) and show in Figure 2 that there are direct synaptic projections from thalamus to dorsal hippocampus. However, this is claim is not supported by any evidence, nor the literature they refer to suggest the existence of such monosynaptic pathway. Instead, the references mentioned here (e.g. Wang et al. 2020) as well as other body of work indicate that whisker information might be relayed to hippocampus through S1 to entorhinal pathways. Therefore, I think either this statement and the illustrations related to that should be supported by solid published anatomical data, or else, must be modified to represent the common understanding of the existing anatomical pathways for relaying whisker sensory information to hippocampus.

2. There are instances where the authors do not describe the original manuscript in the correct way. For example, in Figure 4A, the authors write:
"Simultaneous neuronal recordings from multiple electrodes placed in the ION under two different conditions: passive stimulation (the mouse head is fixed when it is passed through a sliding aperture) and active stimulation (animal is moving freely) increase neuronal response in the CA1 hippocampal region (Pereira et al., 2007)."
While, in the original article (Pereira et al., 2007), simultaneous recordings were performed from S1 and Hippocampus of rats in two different settings: when head-fixed rats receive passive stimulation of the whiskers, or when freely moving rats performed a whisker-dependent discrimination task. So, neither the recording sites nor the experimental paradigms are not reported correctly in Figure 4A.

Minor comments:
1. As a full figure and large body of text is describing whisker to somatosensory pathways, authors should also mention other identified pathways (e.g. paralemniscal pathway) and cover more recent findings (e.g. El Boustani et al. 2020).

2. Figure 1A should be improved in different ways: first, it resembles closely a previously published review from Diamond et al. 2008. Moreover, the representation of whiskers on different rows are not precise, e.g. the straddlers should be drawn in between the rows; for a better illustration look at Adibi et al. 2019. Thirdly, the description and illustration of micro-vibrissae are misleading. In Figure 1A only the

mystical whiskers (macro-vibrissae) are shown, while the micro-vibrissae (the densely-spaced short whiskers located around the nose) are not shown here. I suggest removing the labels of micro and macro-vibrissae from the figure.

3. The texts on the figures are in general too small and should be enlarged.

4. There is need for language improvement. I suggest authors to use the common accepted terminologies in the field rather using synonyms that changes the essential meanings.

5. Another interesting addition to this mini-review which could complement the topics discussed here would be to cover the emergence of learning-related whisker-related sensory responses in Hippocampus (e.g. Le Merre et al. 2019).

Reviewer #2:

The graphical abstract is well written and the illustrations are presented clearly; it was a pleasure to read. The theme is relevant not just to the whisker community, wider somatosensory community but beyond this. However the net could be cast further. The importance of the pathway highlighted is perhaps less well known and I agree it is critical to highlight so but the graphical abstract might be improved to 1) acknowledge other whisker pathways (through VPM, through POM +others LD maybe?) and 2) suggest and amplify that information is spread far wide across the brain and hypothesise how "pathways" might be integrated to complete the sensory landscape.

1st Author Response Letter

Response to comments from Editors and Reviewers:

Comments from Reviewer 1

We appreciate the comment of the reviewer # 1 who considered that our work provided a timely review with visually appealing figures that clearly summarized the relationship between whiskers and hippocampal functions. However, this reviewer expressed some concerns that we have addressed in the following lines

1. This reviewer #1 (R1) expressed *"The authors claim in the text (e.g. page 3, paragraph 3) and show in Figure 2 that there are direct synaptic projections from thalamus to dorsal hippocampus. However, this is claim is not supported by any evidence... Therefore, I think either this statement and the illustrations related to that should be supported by solid published anatomical data, or else, must be modified to represent the common understanding of the existing anatomical pathways for relaying whisker sensory information to hippocampus.".*

Response: We thank the reviewer for identifying this important issue in our work and we apologize for this misleading. In this revised version, we amended Figure 2 and corrected the figure legend and text. <u>Please see Page 3, paragraph 3, Figure 2 and figure legend 2</u>

2. **R1:** *"There are instances where the authors do not describe the original manuscript in the correct way. For example, in Figure 4A... So, neither the recording sites nor the experimental paradigms are not reported correctly in Figure 4A".*

Response: We sincerely apologize for this mistake and thank the reviewer for identifying it for us. In this revised version, we entirely amended Figure 4 and corrected the descriptions provided in both the figure legend and text. Please see Page 4, paragraph 2, Figure 4 and figure legend 4

3. **R1:** *"As a full figure and large body of text is describing whisker to somatosensory pathways, authors should also mention other identified pathways (e.g. paralemniscal pathway) and cover more recent findings (e.g. El Boustani et al. 2020).*

Response: We appreciate this great suggestion. In this revised version, we redesigned our Figure 1 to show the other neural pathways that have been identified for the whisker-elicited tactile information. Please see figure 2.

4. **R1:** *"Figure 1A should be improved in different ways: first, it resembles closely a previously published review from Diamond et al. 2008. Moreover, the representation of whiskers on different rows are not precise, e.g. the straddlers should be drawn in between the rows; for a better illustration look at Adibi et al. 2019. Thirdly, the description and illustration of micro-vibrissae are misleading. In Figure 1A only the mystical whiskers (macro-vibrissae) are shown, while the micro-vibrissae (the densely-spaced short whiskers located around the nose) are not shown here. I suggest removing the labels of micro and macro-vibrissae from the figure".*

Response: We thank the reviewer for identifying this issue and agree that our original figure misrepresent micro-vibrissae and has some similarity with a previously published. Hence, for this revised version we modified Figure 1, where we depicted the micro vibrissae and amended the locations of the straddlers. Please see figure 1.

5. **R1:** *The texts on the figures are in general too small and should be enlarged.*

Response: Again, we thank the reviewer for identifying this issue for us. In this revised version, we adjusted the font sizes in all our figures.

6. **R1:** *There is need for language improvement. I suggest authors to use the common accepted terminologies in the field rather using synonyms that changes the essential meanings.*

Response: We apologize for this matter and amended all the inappropriate terminologies. Please see page 3, paragraph 2; figure legend 2

7. **R1:** *Another interesting addition to this mini-review which could complement the topics discussed here would be to cover the emergence of learning-related whisker-related sensory responses in Hippocampus (e.g. Le Merre et al. 2019).*

Response: We appreciate this great suggestion. In this revised version, we included those outstanding findings. Please see page 5, paragraph 1.

Comments from Reviewer 2

We appreciate the very gentle comments of Reviewer #2 about our work, who stated that this Graphical Review is relevant not just to the whisker community, wider somatosensory community but beyond this. However, this reviewer expressed two concerns that we have addressed in the following lines

1. This reviewer #2 (R2) expressed *"…1) the graphical abstract might be improved to 1) acknowledge other whisker pathways (through VPM, through POM +others LD maybe)?".*

Response: We appreciate this constructive suggestion. In this revised version, we modified Figure 1 and its legend and describe the pathways that relay in several other brain regions. Please see the Graphical Abstract, figure 2 and figure legend 2

1. **R2:** *"2) suggest and amplify that information is spread far wide across the brain and hypothesise how "pathways" might be integrated to complete the sensory landscape".*

Response: R2 concurred with R1 in this topic and, again, we appreciate these great suggestions. To address this comment, we included more information and described the integrative pathways related to tactile processing. Please see Figure 2, Figure 4, page 5, paragraph 1.

Finally, we thank the reviewers for their time and feedback provided in our work.

Accept Letter

Dear Dr. Gonzalez-Perez,

Thank you for submitting your manuscript to Current Research in Neurobiology.

I am pleased to inform you that your manuscript has been accepted for publication. I would, however, request the authors to take reviewer 1's comments for minor edit of figure 2.

My comments, and any reviewer comments, are below.

Your accepted manuscript will now be transferred to our production department. We will create a proof which you will be asked to check, and you will also be asked to complete a number of online forms required for publication. If we need additional information from you during the production process, we will contact you directly.

We appreciate and value your contribution to Current Research in Neurobiology. We regularly invite authors of recently published manuscript to participate in the peer review process. If you were not already part of the journal's reviewer pool, you have now been added to it. We look forward to your continued participation in our journal, and we hope you will consider us again for future submissions.

CRNEUR aims to be a unique, community-led journal, as highlighted in the Editorial Introduction. As part of this vision, we will be regularly seeking input from the scientific community and encourage you and your co-authors to take the survey.

Kind regards,

Abhishek Banerjee
Associate Editor
Current Research in Neurobiology

Editor and Reviewer comments:

Reviewer #1: Authors have addressed most of the raised concerns, and both figures and text have been improved extensively. However, I still have one suggestion:

Figure 2 has been improved compared to the original submission, and authors have now drawn different parallel pathways of information from whiskers to S1 and S2 cortex. However, I still think this figure should be improved in different ways:

a) The lower part of the figure relating to Hippocampus is not accurate. This panel as it is now, implies that whisker information that eventually arrives at Hippocampus is specifically relayed through the lemniscal pathway. There is no reason that this should be the case. Indeed, any information that arrives at S1 (through lemniscal or paralemniscal pathway) could potentially be relayed to Hippocampus; so this panel and the related text in Page 3, paragraph 3 should be modified.

b) The orange (whisker to brainstem) and brown (paralemniscal) colors are hardly indistinguishable.

After, addressing this issue, I recommend the manuscript for publication.

Reviewer #2: Thank you for addressing my comments. I approve the manuscript in its current form.

-------- *End of Review Comments* --------
